# A comparative study of patient satisfaction about anesthesia with dexmedetomidine for ambulatory dental surgery

**DOI:** 10.1186/s13104-022-06246-2

**Published:** 2022-12-22

**Authors:** Levin Garip, Jasmin Verbist, Hendrik Stragier, Joeri Meyns, Dieter Mesotten, Joris Vundelinckx

**Affiliations:** 1 Department of Anesthesiology, Intensive Care Medicine, Emergency Medicine and Pain Therapy, Ziekenhuis Oost-Limburg, Genk, Belgium; 2 CARIM School for Cardiovascular Diseases, Faculty of Health, Medicine and Life Sciences, Maastricht University, Maastricht, Netherlands; 3 Department of Stomatology, Ziekenhuis Oost-Limburg, Genk, Belgium; 4 Faculty of Medicine and Life Sciences, UHasselt, Diepenbeek, Belgium; 5 Critical Department, Ziekenhuis Oost-Limburg, Schiepse Bos 6, 3600 Genk, Belgium

**Keywords:** Dexmedetomidine, Dental surgery, Ambulatory surgery, Patient satisfaction, Study, Monitored anesthesia care, General anesthesia

## Abstract

**Objective:**

Intranasal administration of dexmedetomidine for monitored anesthesia care (MAC) appears to be an effective, safe, and appropriate alternative to general anesthesia (GA) for ambulatory dental surgery. Based on the available evidence we evaluated a new MAC protocol with intranasal dexmedetomidine as the primary choice.

To assess a difference in patient satisfaction and anesthesia-related discomfort between GA and MAC in ambulatory dental surgery, a study was conducted among patients undergoing various dental procedures. Patient satisfaction and anesthesia-related discomfort were assessed on the first postoperative day using the Bauer patient satisfaction questionnaire.

**Results:**

Although the differences were small, patients in the MAC group were overall more satisfied with the general care compared to the GA group (p < 0.02). Patients in the MAC group reported more postoperative drowsiness compared to the GA group (p < 0.05), but less postoperative hoarseness and sore throat (p = 0.005 and p < 0.001, respectively). Moreover, postoperative thirst was more common in the GA group (p = 0.002).

In conclusion, the differences in patient satisfaction and anesthesia-related discomfort between GA and MAC in this implementation study were small but appeared to favor MAC with intranasal dexmedetomidine over GA as anesthesia method during dental ambulatory surgery.

**Supplementary Information:**

The online version contains supplementary material available at 10.1186/s13104-022-06246-2.

## Introduction

Many surgical dental procedures are performed in an ambulatory setting under general anesthesia (GA), monitored anesthesia care (MAC), or with local anesthesia only. GA ensures complete amnesia and hypnosis during the procedure but requires nasotracheal intubation and prolonged recovery time, whereas MAC does not require intubation and usually results in a faster recovery [1]. MAC has the major advantage of relieving a patient's stress in anticipation of the procedure but requires the patient's cooperation during the procedure.

A potential disadvantage of MAC is a compromised airway during the procedure. Titrated administration of anesthetic drugs is therefore paramount, keeping nasopharyngeal reflexes intact. Dexmedetomidine is a promising agent in this regard [2],

Reports about the use of dexmedetomidine for MAC in dental surgery in adult patients mainly compared the combination of the intravenous formula of dexmedetomidine and various short- and long-acting drugs with common hypnotics (e.g., midazolam, fentanyl, remifentanil, ketamine) [3–5]. Overall, the use of dexmedetomidine offered better sedation and better postoperative analgesia compared to midazolam [6, 7].

Intranasal administration of dexmedetomidine appeared to be a suitable option because both the agent’s intranasal and intravenous administration were considered effective, safe, and appropriate for MAC in third molar extractions [7]. Therefore, based on the available evidence, we implemented in our center a new MAC protocol with intranasal dexmedetomidine as the primary choice.

A study was conducted among patients who underwent ambulatory dental surgery with either GA or MAC with dexmedetomidine to assess whether MAC yields a higher patient satisfaction, faster ambulation, and less anesthesia-related discomfort compared to GA. Patients were not randomized because patient preference and motivation to choose a particular anesthesia method have an important effect on their cooperation during the dental procedure and its success. In addition, the surgeon may prefer to perform some procedures under GA.

## Main text

### Materials and methods

#### Study design

A prospective observational trial was performed at Ziekenhuis Oost-Limburg, a tertiary medical center in Belgium. Patients undergoing ambulatory dental surgery between 24 June 2021 and 20 September 2021 were asked to participate in the study during the preoperative anesthesia consultation. Written informed consent was obtained from each patient. Patients were free to choose between GA or MAC in consultation with their surgeon. The study was approved by the local Ethical Committee (File Z-2021038).

#### Inclusion and exclusion criteria

Inclusion criteria were patients undergoing ambulatory dental surgery, eligible for either MAC or GA and who were  ≥ 15 years of age. Exclusion criteria were mental disabilities or a language barrier.

#### Procedure

GA consisted of propofol induction (1–2 mg/kg), remifentanil infusion (0.15 μg/kg/min) and sevoflurane/nitrous oxide maintenance (minimal alveolar concentration 0.8) followed by nasotracheal intubation. During GA, the surgeon applied local anesthesia with Septanest (40 mg/ml articaine, 10 µg/ml adrenaline).

MAC was provided with dexmedetomidine intranasally (1 µg/kg) around 30 min before surgery and by bolus infusion of propofol (10–50 mg) and fentanyl (50–75 µg) titrated by the anesthetist in anticipation of local infiltration by the surgeon.

Paracetamol and ibuprofen were administered orally 30 min before surgery and prescribed postoperatively for both procedures unless contraindicated. Standard antiemetic prophylaxis consisted of intravenous dexamethasone and ondansetron. Patients undergoing GA were instructed not to eat or drink anything on the day of surgery until recovery from the anesthesia. Patients undergoing MAC were instructed to drink 200 mL of a commercially available isotonic fluid on the morning of surgery.

#### Outcomes

The primary outcome was satisfaction on an ordinal 4-point scale with the whole procedure of undergoing GA versus MAC for ambulatory dental surgery on the first postoperative day. Secondary outcomes were anesthesia-related discomfort such as drowsiness, pain at the site of surgery, thirst, hoarseness, sore throat, nausea, or vomiting, feeling cold, confusion or disorientation, pain at the anesthetic injection site, and shivering.

#### Data collection

Patient satisfaction and anesthesia-related discomfort were assessed using the Bauer patient satisfaction questionnaire [8] supplemented with additional questions (see Supplementary materials).

The additional questions assessed the perceived speed of outpatient treatment, potential intraoperative recall, and the presence and nature of intraoperative dreams. Patients were also asked about the worst element of the procedure; whether they drank anything before the procedure, whether they received the anesthesia type they had expected, and whether they had chosen their anesthesia type themselves.

#### Data analysis and statistical methods

We performed univariate analyses to detect differences between groups using Chi-square tests for binary outcome parameters and Mann Whitney U tests for ordinal outcome data. In addition, ordinal and binary logistic regression analyses were performed to correct for baseline characteristics and type of surgery in all outcomes. All assumptions for the binary logistic regression analyses were met. The assumption of proportional odds was not met in all ordinal logistic regression analyses. An α-level of 5% was used in all analyses. All statistical analyses were performed with IBM SPSS Statistics software version 28.0.0 [9].

## Results

### Participants and baseline characteristics

A total of 314 patients for whom ambulatory dental surgery was planned were eligible for the study; of these, 299 patients were included, and 15 were excluded. The anesthesia method the patients chose was GA by 58% and MAC by 42%. After the procedure, 81.6% patients completed the questionnaire. From the GA group, 17% and from the MAC group, 21% did not complete the questionnaire. A flow diagram of the study is depicted in Additional file 1: Figure S1.

The baseline characteristics were compared between the groups, and the gender distribution did not differ (p = 0.4, Additional file 1: Table S1). The mean age of the patients in the GA group (24.7 years, [SD] ± 14.2) was lower than that of the patients in the MAC group (33.5 years, SD ± 19.1) (p = 0.005, Additional file 1: Table S1). Those in the GA group underwent fewer third molar extractions (n = 115, 79.9%) than in the MAC group (n = 66, 66%), as the latter underwent more primarily implant surgery and other extractions (p = 0.03, Additional file 1: Table S1).

### Patient satisfaction with the procedure

**Fig. 1:**
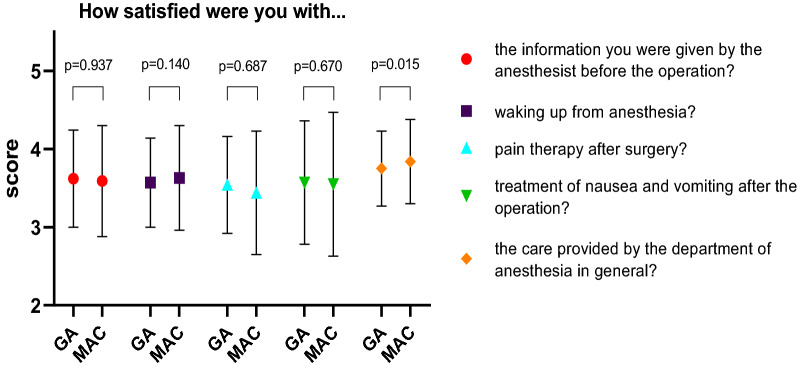
Patient satisfaction. Mean patient satisfaction scores in both groups and their respective p-values of the Mann–Whitney U test. Descriptive values were converted to numerical values. “Very dissatisfied” equals 1, “dissatisfied” equals 2, "satisfied" equals 3, and “very satisfied” equals 4. GA, general anesthesia; MAC, monitored anesthesia care

All mean satisfaction scores ranged between “satisfied” (score 3) and “very satisfied” (score 4) in both groups (Fig. 1). Patients in the MAC group were, however, more satisfied (mean score 3.84) with the care provided by the department of anesthesia in general compared to the GA group (mean score 3.75) (p = 0.015). Patients were equally satisfied in both groups regarding the information given before the operation ((p = 0.937), emergence from anesthesia (p = 0.140), analgetic (p = 0.687), and treatment of nausea and vomiting (p = 0.670) (Additional file 1: Table S2, Fig. 1).

### Anesthesia-related discomfort

**Fig. 2:**
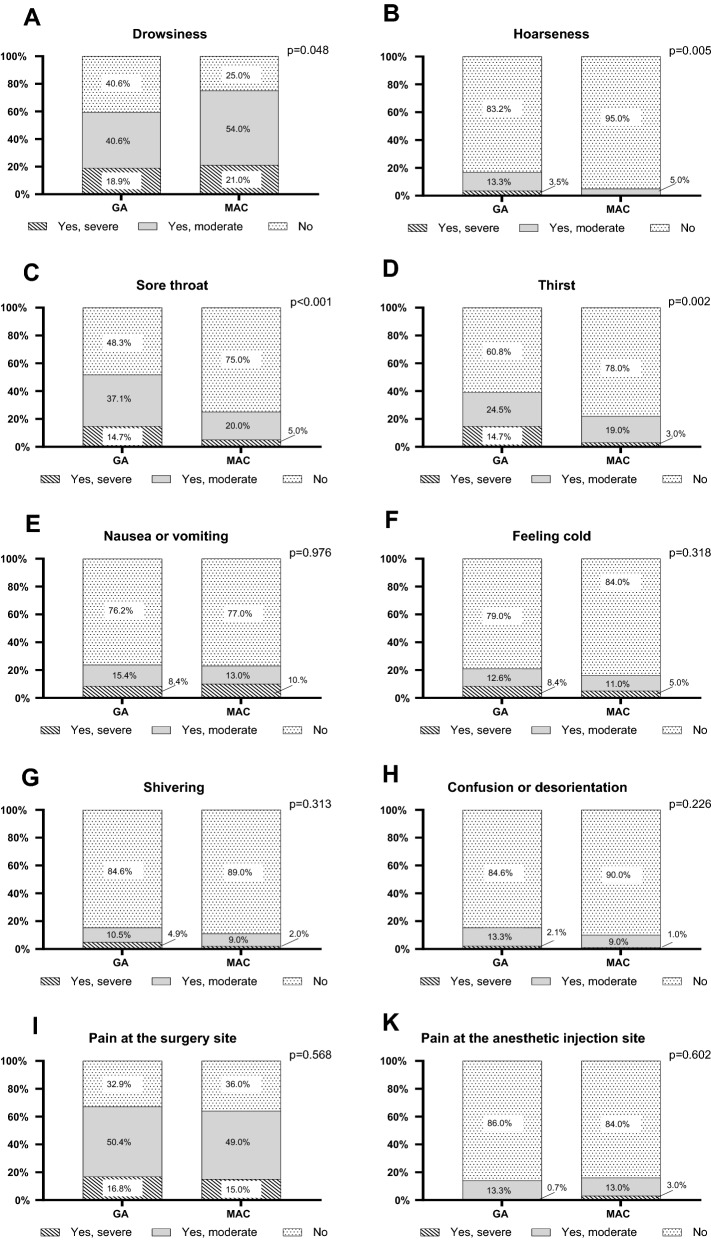
Severity of the various types of anesthesia-related discomfort. The question ‘At any stage after your operation have you had…’ could be answered with ‘Yes, severe’, ‘Yes, moderate’, or ‘No’. The types of anesthesia-related discomfort were: **A** drowsiness; **B**, hoarseness, **C**, sore throat; D, thirst; **E**, nausea or vomiting; **F**, feeling cold; **G**, shivering; **H**, confusion or disorientation; **I**, pain at the site of surgery; **K**, pain at the site of anesthetic injection. The p-values of the Mann–Whitney U tests comparing both groups are shown. GA, general anesthesia; MAC, monitored anesthesia care

When asked about anesthesia-related discomfort, 75% of patients in the MAC group reported more severe or moderate drowsiness compared to the 59% in the GA group (p = 0.048, Fig. 2A). In contrast, patients in the GA group had more postoperative hoarseness and sore throat than those in the MAC group (p = 0.005 and p < 0.001, respectively) (Fig. 2B and C). Also, postoperative thirst was more common in the GA group, with 15% of the GA group reporting severe thirst compared to 3% in the MAC group (p = 0.002) (Fig. 2D). All results of the univariate and multivariate analyses are shown in Additional file 1: Table S3.

### The worst aspect of the procedure

When asked what the worst aspect of the anesthetic procedure was, the most common answer was 'fear' in both groups, as 31% of patients in the GA group and 27% in the MAC group reported this. This was followed by 'not being able to do daily activities' in 22% of the GA group, and 'the recovery process' in 21% of the MAC group. (Additional file 1: Figure S2).

### Awareness during the procedure

**Fig. 3:**
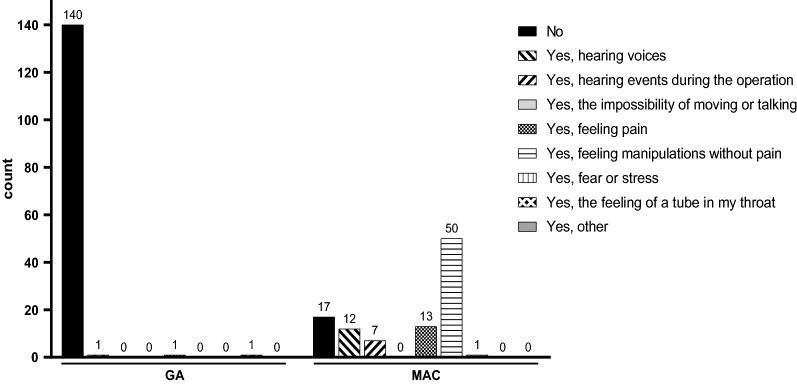
Intraoperative recall. The number of patients' selected responses regarding intraoperative recall for both groups. The question asked was ‘Can you remember anything from the period between falling asleep and awakening?’ GA, general anesthesia; MAC, monitored anesthesia care

When asked whether the patients could remember anything from the period between falling asleep and awakening, there was a notable difference between the two groups. Almost everyone (n = 140, 98%) in the GA group had complete amnesia as they remembered nothing, while this was only 17% in the MAC group. In the MAC group, 50% reported painless sensations, and 13% of the patients perceived pain. However, only one of the patients undergoing MAC reported fear or stress during the procedure (Fig. 3).

## Discussion

Due to the non-randomized nature of our study, a difference in type of surgery performed and age was observed. All the participants were given the choice between GA or MAC without any obligations by the surgeon or anesthetist. The complexity of the procedure did not influence the decision making as all the procedures included in this study could be technically well performed using both GA or MAC. If there are no significant risks connected to the patient’s history to undergo the procedure under GA or MAC, we leave the choice up to the patients. Nevertheless, an adjustment for possible confounders like type of surgery and age was made when using multivariate analysis, thereby eliminating any potential bias in our results.

In multiple hospitals, there is a standardized protocol for using MAC in dental surgery. Patients are given the choice between MAC or GA because some patients do not prefer the level of awareness accompanied by MAC. The scope of this study is to compare our MAC protocol with the use of general anesthesia in order to elucidate the benefits and disadvantages of both with the aim of informing our patients more accurately in the future. All the procedures included in this study could be technically well performed using both GA or MAC. Personal preference did play a role in participant allocation. However, it was not considered expedient to randomize since patient's motivation and cooperation during the MAC procedure is crucial for success.

This prospective observational trial shows that the differences in patient satisfaction between patients undergoing MAC or GA for ambulatory dental surgery are small, although the MAC group was significantly more satisfied in general with the care provided by the department of anesthesia. In addition, anesthesia-related discomfort was different between the groups for four of the eight signs of discomfort analyzed, and most of these were in favor of MAC. Based on these results the new MAC protocol with intranasal dexmedetomidine for ambulatory dental surgery appears well tolerated by the patients.

A systematic review of seventeen randomized clinical trials on conscious sedation techniques for third molar surgery found that, although findings were inconclusive about the role of conscious sedation for managing dental anxiety, six studies reported that conscious sedation was associated with an improvement of dental anxiety [10]. We emphasize the importance of adequately informing the patient about the procedure beforehand. As MAC is a conscious sedation technique it may be more appropriate for patients with dental anxiety. Patients in the GA group felt more autonomous in choosing the type of anesthesia and were more likely to receive the anesthesia they expected. This indicates a possible lack of information or an insufficiently clear image for patients of what MAC is about.

MAC in ambulatory dental surgery leads to a risk of compromising the airway. However, our results clearly show less postoperative hoarseness and sore throat in these patients. Nasotracheal intubation, in our center done without administration of muscle relaxants, may have caused the higher incidence of the latter [11].

The MAC patients were allowed to drink an isotonic solution until 1 h before the procedure, while GA patients were instructed not to eat or drink anything before the procedure. This resulted in a less pronounced thirst in MAC patients and may have help with a faster postoperative recovery [12].

When performing MAC in ambulatory dental surgery, one should ensure adequate local anesthetic infiltration since 13% of our patients reported pain.Most patients who underwent MAC have a recall of painless sensations, surgical manipulations, or hearing voices. These sensations may not be negative for patients but should be clearly communicated in the preoperative counseling for a successful MAC procedure.

## Conclusions

In conclusion, MAC seems promising for the use in ambulatory dental surgery with faster recovery times, fewer anesthesia-related complications, and a high patient satisfaction after the procedure. Adequate and complete preoperative counseling is mandative to reduce preoperative fear and stress. The use of intranasal dexmedetomidine may lead to a prolonged postoperative drowsiness but may be useful in patients with severe anxiety. The instruction to drink an isotonic fluid up to 1 h before the procedure gives the patient more comfort and less thirst afterwards. It also avoids the anesthesia-related complications like hoarseness and a sore throat after nasogastric intubation. We can conclude that MAC based on intranasal dexmedetomidine is a promising alternative for GA in dental ambulatory surgery.

### Limitation section

First, this study was not randomized nor blinded. Patients were free to choose their desired type of anesthesia in consultation with their surgeon. In addition, a multiple regression analysis was performed to correct for gender, age, and type of surgery. However, there may also be other patient-related or surgery-related characteristics that were not accounted for which may confound the results. Furthermore, the administered anesthesia in both groups was radically different. Other instructions were given, and other medications were used in both groups. This makes it hard to determine the true causality of the results.

The subjective nature of the outcome measures was another limitation of this study. Despite the use of a validated questionnaire, namely the Bauer patient satisfaction questionnaire, these data must be interpreted with caution as these study data are not objective measures. Another problem is the possible introduction of recall bias as all questionnaires were filled in at least 1 day after surgery. In some cases, patients were contacted a few days later because they were unavailable on the postoperative day. Regarding the statistical analyses, some regression analyses had a low model fit or did not meet the required assumptions, which means that the statistical outcomes of these analyses must be interpreted with caution.

## Supplementary Information

**Additional file 1: Table S1. **Baseline characteristics. **Table S2.** Survey results on patient satisfaction. **Table S3.** Regression analyses. **Figure S1.** Consort diagram. **Figure S2**. Main discomfort during or after the procedure.

## Data Availability

The datasets used and analyzed during the current study are not available publicly due to privacy reasons, but are available from the corresponding author on reasonable request.

## Abbreviations

EudraCT: European Union drug regulating authorities clinical trials database
GA: General anesthesia
MAC: Monitored anesthesia care
OR: Odds ratio
SD: Standard deviation
