# Supplementary material for: A comparative study of patient satisfaction about anesthesia with dexmedetomidine for ambulatory dental surgery
Source: BMC Res Notes. 2022 Dec 22;15:376. doi: 10.1186/s13104-022-06246-2 (PMC9773427; doi:10.1186/s13104-022-06246-2)
Supplement: Supplementary file 1 — Additional file 1: Table S1. Baseline characteristics. Table S2. Survey results on patient satisfaction. Table S3. Regression analyses. Figure S1. Consort diagram. Figure S2. Main discomfort during or after the procedure. [file 13104_2022_6246_MOESM1_ESM.docx]

Questionnaire

First and last name:

________________________________________________________________

Date of birth (day/month/year):

________________________________________________________________

Date of the procedure (day/month/year):

________________________________________________________________

Which type of anesthesia did you receive?

- General anesthesia
- Sedation (monitored anesthesia care)

At any stage after your operation have you had the following?

|  | No | Yes, moderate | Yes, severe |
| --- | --- | --- | --- |
| Drowsiness |  |  |  |
| Pain at the site of surgery |  |  |  |
| Thirst |  |  |  |
| Hoarseness |  |  |  |
| Sore throat |  |  |  |
| Nausea or vomiting |  |  |  |
| Feeling cold |  |  |  |
| Confusion or disorientation |  |  |  |
| Pain at the site of the anaesthetic injection |  |  |  |
| Shivering |  |  |  |

How satisfied were you with...

| the information you were given by the anaesthesist before the operation? | 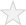 | 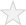 | 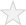 | 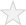 |
| --- | --- | --- | --- | --- |
| waking up from anaesthesia? | 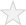 | 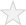 | 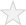 | 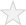 |
| pain therapy after surgery? | 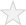 | 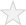 | 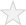 | 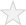 |
| treatment of nausea and vomiting after the operation? | 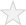 | 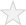 | 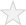 | 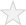 |
| the care provided by the department of anaesthesia in general? | 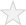 | 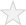 | 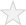 | 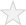 |

Would you recommend this anesthetic service to friends and family?

- Yes
- No

Did you expect to receive a general anesthesia for this procedure?

- Yes
- No

Did you autonomously choose your type of anesthesia for this procedure?

- Yes, I chose for this type of anesthesia myself
- No, my surgeon recommended this type of anesthesia

What is the last thing you could remember before falling asleep?

- Being in the preoperative room
- Being with family
- The sensation of the face mask
- A burning or stinging sensation from the infusion
- Being in the operation room
- Hearing voices
- The scent of a gas
- Not applicable
- Other ________________________________________________

What is the first thing you could remember when waking up?

- Hearing voices
- The sensation of a tube in my throat
- Being with family
- Feeling pain
- The sensation of the face mask
- Being in the recovery room
- Being in the operation room
- Being at the intensive care
- Nothing
- Not applicable
- Other ________________________________________________

Can you remember anything from the period between falling asleep and awakening?

- No
- Yes, hearing voices
- Yes, hearing events during the operation
- Yes, the impossibility of moving or talking
- Yes, feeling pain
- Yes, feeling manipulations without pain
- Yes, fear or stress
- Yes the feeling of a tube in my throat
- Yes, other ________________________________________________

Did you dream during the procedure?

- Yes ________________________________________________
- No

Were your dreams disturbing?

- Yes
- No
- I had no dreams

What was the worst about the procedure?

- Fear
- The recovery process
- Awareness of the procedure
- Feeling pain
- Not being able to do daily activities
- Nothing
- Other ________________________________________________

Did you drink something before the procedure?

- Yes
- No

Do you have the feeling you could go home quickly after the procedure (smooth progress from arrival to departure)?

- Yes
- No

Tables

# Table S1 Baseline characteristics

**Distribution of type of surgery**

|  |  |  | General anesthesia | Monitored anesthesia care | Total |
| --- | --- | --- | --- | --- | --- |
| Type of surgery | Wisdom tooth extractions | Count | 115 | 66 | 181 |
|  |  | % | 79.9% | 66.0% | 74.2% |
|  | Other extractions | Count | 15 | 23 | 38 |
|  |  | % | 10.4% | 23.0% | 15.6% |
|  | Implant surgery | Count | 6 | 7 | 13 |
|  |  | % | 4.2% | 7.0% | 5.3% |
|  | Other | Count | 8 | 4 | 12 |
|  |  | % | 5.6% | 4.0% | 4.9% |
| Total |  | Count | 144 | 100 | 244 |
|  |  | % | 100.0% | 100.0% | 100.0% |

**Gender distribution**

|  |  |  | General anesthesia | Monitored anesthesia care | Total |
| --- | --- | --- | --- | --- | --- |
| Gender | Male | Count | 57 | 45 | 102 |
|  |  | % | 39.6% | 45.0% | 41.8% |
|  | Female | Count | 87 | 55 | 142 |
|  |  | % | 60.4 % | 55.0% | 58.2% |
| Total |  | Count | 144 | 100 | 244 |
|  |  | % | 100.0% | 100.0% | 100.0% |

**Age distribution**

|  |  | Count | Mean | Std. Deviation | Std. Error Mean |
| --- | --- | --- | --- | --- | --- |
| Age | General anesthesia | 144 | 24.70 | 14.2020 | 1.183 |
|  | Monitored anesthesia care | 100 | 33.50 | 19.129 | 1.913 |

## Table S2 Survey results on patient satisfaction

|  | **GA, n = 143** | |  | **MAC, n = 100** | |
| --- | --- | --- | --- | --- | --- |
| **Survey question** | **Mean (SD)** | **range** |  | **Mean (SD)** | **range** |
| How satisfied were you with... |  |  |  |  |  |
| the information you were given by the anesthetist before the operation? | 3.62 (0.62) | 1-4 |  | 3.59 (0.71) | 1-4 |
| waking up from anaesthesia? | 3.57 (0.57) | 2-4 |  | 3.63 (0.67) | 1-4 |
| pain therapy after surgery? | 3.54 (0.62) | 1-4 |  | 3.44 (0.79) | 1-4 |
| treatment of nausea and vomiting after the operation? | 3.57 (0.79) | 0-4 |  | 3.55 (0.92) | 1-4 |
| the care provided by the department of anesthesia in general? | 3.75 (0.48) | 2-4 |  | 3.84 (0.54) | 1-4 |

n, number; SD, standard deviation

## Table S3 Regression analyses

|  | | **Univariate analysis^a^** | **Multivariate analysis^b^** | | | | | |
| --- | --- | --- | --- | --- | --- | --- | --- | --- |
| **Survey question** | | **p-value** | **Model information** | | | | | **p-value** |
|  |  |  | **Model fitting** | **Goodness-of-fit** | | **Assumption of equal variances** | |  |
| **At any stage after your operation have you had the following?** | |  |  |  | |  | |  |
|  | Drowsiness | 0.048 | Good | Good | | Met | | 0.002 |
|  | Pain at the site of surgery | 0.568 | Bad | Good | | Met | | 0.760 |
|  | Thirst | 0.002 | Good | Good | | Met | | 0.002 |
|  | Hoarseness | 0.005 | Good | Good | | Broken | | 0.009 |
|  | Sore throat | <0.001 | Good | Good | | Met | | <0.001 |
|  | Nausea or vomiting | 0.976 | Bad | Good | | Broken | | 0.827 |
|  | Feeling cold | 0.318 | Bad | Good | | Met | | 0.560 |
|  | Confusion or disorientation | 0.226 | Bad | Good | | Broken | | 0.589 |
|  | Pain at the site of the anesthetic injection | 0.602 | Bad | Good | | Met | | 0.369 |
|  | Shivering | 0.313 | Bad | Good | | Met | | 0.732 |
| **How satisfied were you with…** | |  |  |  | |  | |  |
|  | the information you were given by the anesthetist before the operation? | 0.937 | Bad | Bad | | Met | | 0.863 |
|  | waking up from anaesthesia? | 0.140 | Good | Bad | | Broken | | 0.340 |
|  | pain therapy after surgery? | 0.687 | Good | Good | | Met | | 0.215 |
|  | treatment of nausea and vomiting after the operation? | 0.670 | Bad | Good | | Broken | | 0.827 |
|  | the care provided by the department of anesthesia in general? | 0.015 | Good | Bad | | Broken | | 0.006 |
|  | | | | | | | | |
|  | | **Chi-square test** | | | **Binary regression analysis** | | | |
| **Survey question** | | **p-value** | **Odds ratio** | | **p-value** | | **Odds ratio** | |
| Would you recommend this anesthetic service to friends and family? | | 0.332 | 1.731 | | 0.718 | | 1.253 | |
| Did you receive the type of anesthesia you expect for this procedure? | | <0.001 | 4.467 | | 0.001 | | 4.106 | |
| Did you autonomously choose your type of anesthesia for this procedure? | | <0.001 | 4.488 | | <0.001 | | 4.155 | |
| Do you have the feeling you could go home quickly after the procedure (smooth progress from arrival to departure)? | | 0.001 | 3.344 | | 0.006 | | 2.950 | |
| Did you dream during the procedure? | | <0.001 | N/A | | 0.996 | | N/A | |

^a^Mann Whitney U test . ^b^Ordinal regression analysis. N/A, not applicable

## Figure S1 Consort diagram


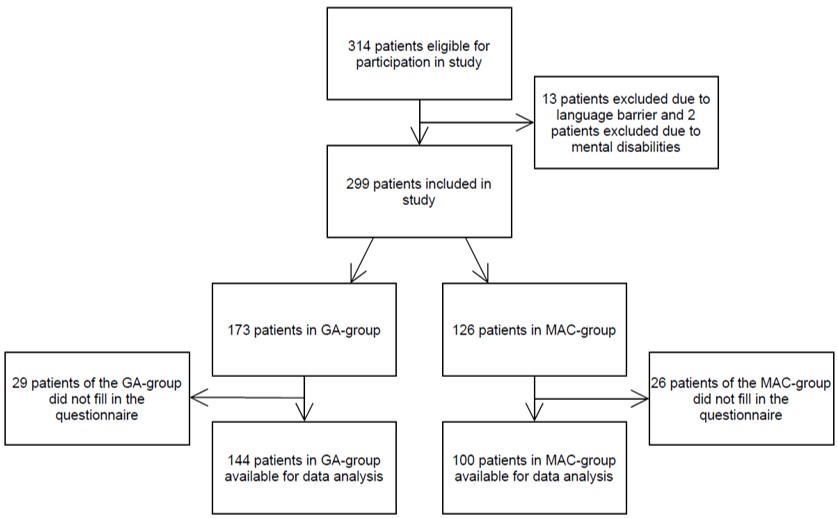


## Figure S2 Main discomfort during or after the procedure
